# Advances in the Therapeutic Use of Non-Ergot Dopamine Agonists in the Treatment of Motor and Non-Motor Symptoms of Parkinson’s Disease

**DOI:** 10.2174/1570159X20666220915091022

**Published:** 2023-04-12

**Authors:** Xiao-Zhong Jing, Hui-Jia Yang, Reyisha Taximaimaiti, Xiao-Ping Wang

**Affiliations:** 1 Department of Neurology, Jiading Branch of Shanghai General Hospital, Shanghai Jiao Tong University, School of Medicine, Shanghai, China;; 2 Department of Neurology, TongRen Hospital, Shanghai Jiao Tong University, School of Medicine, Shanghai, China;; 3 Center for Clinical Research on Neurological Diseases, The First Affiliated Hospital, Dalian Medical University, Dalian 116021, China

**Keywords:** Advances, dopamine (DA) agonists, motor symptoms, non-motor symptoms, Parkinson's disease, treatment

## Abstract

Dopamine (DA) agonists, as an excellent dopamine replacement therapy for patients with early and advanced Parkinson's disease (PD), play a vital role in controlling motor and several non-motor symptoms. Besides, the application of DA agonists may delay levodopa therapy and the associated risk of motor complications. Indeed, each DA agonist has unique pharmacokinetic and pharmacodynamic characteristics and therefore has different therapeutic efficacy and safety profile. The comorbidities, significant non-motor manifestations, concomitant medications, and clinical features of PD individuals should guide the selection of a specific DA agonist to provide a more patient-tailored treatment option. Thorough knowledge of DA agonists helps clinicians better balance clinical efficacy and side effects. Therefore, this review refers to recent English-written articles on DA agonist therapy for PD patients and summarizes the latest findings on non-ergot DA agonists as well as the advantages and disadvantages of each compound to help clinicians in the selection of a specific DA agonist. In addition, novel D1/D5 partial agonists and new formulations of DA agonists are also discussed.

## 1 INTRODUCTION

Parkinson's disease (PD) is a progressive neurodegenerative disorder, affecting 1% of the population aged > 60 years [1]. The pathophysiological feature of PD is a progressive loss of dopaminergic neurons in the pars compacta of the substantia nigra, leading to dopamine deficiency in the striatum [2]. The main clinical manifestations of PD include bradykinesia, resting tremor, rigidity, and postural instability. In addition, most PD individuals also experience various non-motor symptoms, including cognitive impairment, sleep disturbance, mental disorder, and autonomic nerve dysfunction [3].

The primary cause of dopamine neurodegeneration in PD remains unclear, but there is evidence that it may be multifactorial in terms of both etiology and pathogenesis [1]. The complex interaction between genes and environment determines the development of PD, and the presence of multiple pathways involving genes, neural networks, organelles, and proteins may result in the heterogeneity of symptom presentation [4]. Oxidative damage, neuroinflammation, mitochondrial dysfunction, and abnormal protein aggregation may play a significant role in the pathogenesis of PD [5]. Once these processes are initiated, they will continue to cause damage to dopaminergic neurons and have a negative impact on the efficacy of dopamine replacement therapy.

### 1.1 Current Treatment of PD

Dopamine replacement with levodopa remains the most effective therapeutic option for the cardinal features of PD. Although levodopa has profound benefits for PD sufferers, the duration of its effects decreases over time, known as wearing-off. Certain features may become insufficiently responsive to levodopa, with rapid switching between time spent by PD sufferers in a state of mobility (ON-time) and immobility (OFF-time) [6]. Levodopa-induced dyskinesia (LID), characterized by abnormal involuntary muscle movements, may also occur due to the long-time use of levodopa [6].

Currently, treatment for movement fluctuations and LID includes a combination of controlled-release levodopa with the addition of catechol-O-methyltransferase inhibitors or monoamine oxidase B inhibitors or the use of high doses of amantadine [7]. However, these therapeutic medications do not completely resolve levodopa-related motor complications. In addition, although deep brain stimulation can be effective in improving motor complications of PD individuals, these surgical treatments are limited to specific patient populations [7]. How to prevent and relieve motor fluctuations and LID have become important factors to be considered in PD treatment.

### 1.2 Dopamine (DA) Agonists

These issues clearly emphasize the urgent need for alternative therapeutic medication to relieve the symptoms of PD, while reducing the incidence of adverse reactions. Dopamine (DA) agonists may make up for the deficiency of levodopa. DA agonists provide a promising alternative or adjunct to levodopa therapy in PD and are related to a lower risk of motor complications and dyskinesia [8]. Indeed, some clinical trials have also shown that PD patients randomized to start long-acting DA agonists therapy have a lower risk of motor complications than those randomized to short-acting levodopa, even though levodopa is more effective for improving motor symptoms [9-12].

Dopamine (DA) agonists exert their effect mainly by stimulating dopamine receptors in the striatum. Dopamine receptors are widely distributed not only in the central nervous system but also in the periphery. Dopamine receptors can be divided into the D1 family (D1 and D5 receptors) and D2 family (D2, D3, and D4 receptors) according to their biochemical and pharmacological properties [13]. Currently, DA agonists commonly used in clinics mainly target D2 family receptors, especially D2 and D3 receptors. In recent years, DA agonists primarily act on D1/D5 receptors. They are currently undergoing clinical trials.

**Fig. (1):**
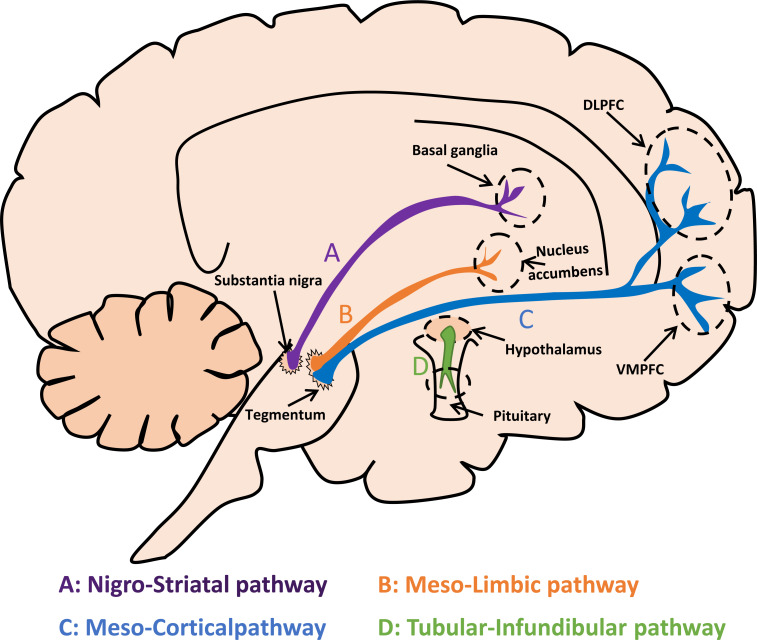
Dopaminergic pathways in the brain. The nigro-striatal pathway projects from substantia nigra to neo-striatum and is primarily involved in the regulation of motor activity (**A**). Meso-limbic pathway projects from ventral tegmental to nucleus accumbens and olfactory nodules, which are primarily involved in the regulation of reward, emotion, and memory (**B**). Executive function is mainly mediated by the Meso-cortical pathway connecting the ventral tegmental to the prefrontal cortex (**C**). The infundibular canal pathway originates from the arcuate nucleus of the hypothalamus and projects to the pituitary gland, and is mainly involved in the regulation of neuroendocrine and waking sleep cycles (**D**). **Abbreviations**: DLPFC: Dorsolateral Prefrontal Cortex; VMPFC: Ventromedial Prefrontal Cortex.

There are four dopaminergic pathways in the central nervous system (Fig. **1**). The nigrostriatal pathway is primarily involved in the regulation of motor activity and is mediated by D1, D2 and D3 receptors [13, 14]. D1 and D2 receptors are highly concentrated in the caudate nucleus and lenticular nucleus. D1 receptors are observed mainly in the internal globus pallidus and substantia nigra pars reticulata (direct pathway), while D2 receptors are primarily presented in the striatal projections to external globus pallidus (indirect pathway) [15, 16]. Compared with D1 receptors, the expression of D5 receptors is limited and only presented in the hippocampus, lateral papillary nucleus, and hypothalamic parafascicular nucleus. D5 receptors are crucial for the regulation of cognition, attention, decision-making, and motor learning [13].

Mesolimbic pathway projects from ventral tegmental to nucleus accumbens and olfactory nodules, preferentially expressing D3 receptors and is primarily involved in the regulation of reward, emotion, addiction, and memory. The extensive expression of D3 receptors in the frontal cortex, limbic system, and thalamus reveals that the D3 receptor is crucial for modifying behavior [17]. Executive function is mediated by the mesocortical pathway connecting the ventral tegmental to the prefrontal cortex, which contains the highest expression of the D4 receptor [13, 15]. Prefrontal cortex dopamine regulates various executive functions dominated by the frontal lobe *via* acting on D1 and D2 receptors [18-20]. The infundibular canal pathway originates from the arcuate nucleus of the hypothalamus and project to the pituitary gland and is mainly involved in the regulation of neuroendocrine and waking sleep cycles [13].

Outside the central nervous system, dopamine receptors can be observed in blood vessels, sympathetic ganglia, postganglionic sympathetic nerves, and kidneys. In kidneys, stimulation of D1 receptors is related to renal vasodilation and an increased electrolyte excretion, which determines the increase of renal flow, diuresis, natriuresis and subsequent hypotension. D2 receptors mediate bradycardia, reduction of afterload and vasodilatation in certain vascular beds, which also determines hypotension [21, 22].

Dopaminergic mechanisms also play a significant role in the modulation of gastrointestinal movement. Dopamine produces a direct relaxation effect by stimulating muscular D2 receptors distributed in the lower esophageal sphincter and stomach. In addition, dopamine exerts indirect inhibitory effects on muscular tissue through the inhabitation of acetylcholine release from intrinsic cholinergic motor neurons by activating prejunctional D2 receptors [23].

Basal ganglia appear to be involved in the regulation of heart rate and blood pressure. It has been shown that the activation of D2/D3 receptors was positively correlated with heart rate variability and had a negative effect on supine systolic blood pressure and heart rate [24]. Activation of postsynaptic dopamine receptors located on vascular smooth muscle also leads to vasodilation and subsequent lower blood pressure [25-27]. However, the stimulation of D2 receptors in the heart results in a decreased heart rate and left ventricular contractility [28].

Conceivably, dopamine receptors participate in regulating various functions, including motor activity, learning, reward, emotion, addiction, memory, cognition, and regulation of neuroendocrine pathways. The wide distribution of dopamine receptors and their involvement in a variety of functional regulations provide the anatomical foundation not only for the curative effects of DA agonist medications but also for their adverse reactions.

### 1.3 DA Agonists Approved for PD and Those Undergoing Clinical Trials

Ten different DA agonists have been approved during the last 4 decades for the management of PD. Five of them are ergot DA agonists (cabergoline, pergolide bromocriptine, lisuride, and dihydroergocryptine). Currently, clinical use of ergot DA agonists, such as bromocriptine, cabergoline, dihydroergocryptine, and pergolide, is no longer advocated due to some serious adverse reactions, including pulmonary fibrosis, ascites, valvular heart disease, pleural effusion, and pericardial effusion [29-32]. Although no link has been found between the application of lisuride and fibrotic reactions [33], the clinical use of lisuride was limited due to mental changes, dyskinesias, and peripheral edema [34].

The five others are non-ergot compounds (apomorphine, rotigotine, pramipexole, ropinirole, and piribedil) that are commonly used for the treatment of PD. Rotigotine, ropinirole, and pramipexole have shown equal clinical efficacy for motor symptoms in PD both as a monotherapy and as adjunctive to levodopa [7]. Apomorphine has been recommended for advanced PD troubled with drug-resistant OFF time and peak dose dyskinesia that cannot be fully controlled by standard oral treatment [7]. Piribedil has been shown to be efficacious as monotherapy and as adjunctive to levodopa for early, non-fluctuating PD individuals [7]. In recent years, several novel D1/D5 partial agonists, including PF-06412562, PF-06649751, and PF-06669571, are also undergoing clinical trials for the management of PD.

In this review, we mainly discuss non-ergot DA agonists, which are known to have beneficial effects for patients with early and advanced PD [7, 35]. In particular, the advantages and disadvantages of each compound in controlling motor and non-motor symptoms will be discussed to help clinicians in the selection of a specific DA agonist. Furthermore, novel formulations of D1/D5 partial agonists and newly emerged post-marketing safety considerations will also be discussed.

## 2 SEARCH STRATEGY

We reviewed English-written articles published in PubMed by using the keywords, such as ‘Parkinson's disease’ and‘Dopamine agonists’ with one of the following: ‘Apomorphine’, ‘Rotigotine’, ‘Pramipexole’, ‘Ropinirole’, ‘Piribedil’, ‘Impulse Control Disorders’, ‘Dopamine Agonist Withdrawal Syndrome’, and ‘Cardiological Adverse Effects’. In most cases, papers were only selected for review when there was an established rating scale or a well-described measurement of endpoints. Systematic reviews and meta-analyses were included if they reported on DA agonist’s treatment for PD. Studies with insufficient data, duplicated publications, and drugs being tested on animals were excluded. A brief overview of the preclinical evidence of DA agonists for the treatment of PD is presented in the following section; however, a full review is beyond the scope of this article.

## 3 PRECLINICAL EVIDENCE OF DA AGONISTS FOR THE TREATMENT OF PD

The beneficial effects of DA agonists on motor impairments have been demonstrated in several experimental animal PD models. Before starting clinical investigations, the above-mentioned DA agonists were all evaluated in animal models of PD to evaluate their antiparkinsonian effects, pharmacokinetic, and toxicological profiles. A brief overview of the published findings from PD animal models of DA agonists will be presented in the following section.

Preclinical studies have found that apomorphine, rotigotine, pramipexole, ropinirole, and piribedil were able to reverse motor impairments in N-methyl-4-phenyl-1,2,3,6-tetrahydropyridine (MPTP) treated primates, reserpine or haloperidol treated rats, and 6-hydroxydopamine (6-OHDA) lesioned rodents [36-43]. In addition, apomorphine and pramipexole have demonstrated neuroprotective effects in MPTP lesioned mice [44, 45]. Recently, a study has reported that the pramipexole transdermal patch provided neuroprotective effects in the MPTP lesioned mice [46]. Another study suggests that rotigotine may have neuroprotective effects [47], but the results were not further assessed in PD animal models or humans.

PF-06412562, PF-06649751, and PF-06669571 are novel compounds with high selectivity for D1/D5 receptors and non-catechol structure. Preclinical studies have shown that non-catechol partial D1 agonists were efficacious in preclinical models of PD [48-50]. In a study on monkeys with MPTP-induced parkinsonism, PF-06669571 demonstrated efficacy for improving scores on observational tests of parkinsonian behaviors [51].

## 4 DA AGONISTS ALREADY APPROVED FOR PD

### 4.1 D1/D2 Family Agonists

#### 4.1.1 Apomorphine

Apomorphine is a broad-spectrum DA agonist that interacts with both D1 family (D1, D5) and D2 family (D2, D3, D4) receptors [52]. Apomorphine is mainly administered by intermittent subcutaneous injection or continuous subcutaneous infusion due to its low oral bioavailability and extensive first-pass metabolism [53]. Intermittent subcutaneous injection of apomorphine has been recommended as a rescue treatment for PD patients troubled with remarkable episodes of wearing off for its rapid onset of action [54]. Intermittent injections of apomorphine have been shown to improve the drug-resistant OFF periods and peak dose dyskinesia in advanced PD patients uncontrolled by standard oral therapy, while the treatment is available only in a handful of European countries [55]. An expert consensus group also recommended that intermittent injection of apomorphine is appropriate for PD patients suffering from both motor and non-motor OFF periods in need of rapid symptom relief [56]. Apomorphine infusion is recommended when standard oral treatment can no longer fully control the OFF period, or when the rescue dose of apomorphine injection is efficacious while needs to be too frequent (over 4-6 times a day), or for whom related to increasing dyskinesia [56]. In 2018, a randomized clinical study involving 106 PD subjects was conducted to estimate the efficacy and tolerability of continuous subcutaneous infusion of apomorphine as first-line therapy in PD patients troubled with persistent motor fluctuations. The study found that continuous infusion of apomorphine significantly reduced OFF time in PD patients compared to placebo, and apomorphine was well tolerated without any significant adverse effects [57].

Up to now, the primary route of administration for apomorphine in PD patients has been subcutaneous, either by intermittent subcutaneous injection or continuous infusion. Due to the adverse reactions of subcutaneous administration, several alternative administration routes have been developed, some of which are under active clinical trials. An inhaled apomorphine powder (VR040) has been developed and tested in a phase IIa study of 24 PD individuals who suffered from motor fluctuations. The study found that inhaled apomorphine was well tolerated but of limited benefit [58]. Two larger clinical trials with a total of 102 PD individuals found that inhaled apomorphine showed great promise as an alternative to intermittent subcutaneous injection, and the UPDRS-III score was significantly improved in the inhaled apomorphine treatment group. However, inhalation of apomorphine failed to significantly reduce daily OFF time [59, 60]. These studies found no pulmonary safety issues, but no further research was conducted on VR040 for the treatment of PD.

In 2016, a phase II trial involving 19 PD patients found that a new apomorphine sublingual film can rapidly and effectively convert a patient from the OFF state to the ON state as first-line therapy. Of the 19 PD subjects, 15 PD individuals achieved a full ON state within 30 minutes [61]. In 2020, a phase III study involving 109 PD individuals was designed to assess the safety and effectiveness of apomorphine sublingual film as first-line therapy in the treatment of OFF episodes in PD sufferers. Although nearly one-third of PD subjects discontinued treatment mainly due to oropharyngeal adverse reactions, apomorphine sublingual film provided an effective, on-demand treatment for OFF episodes for the majority of PD subjects in the study, with common adverse effects, including daytime somnolence and nausea [62]. An open-label study on 141 PD patients with OFF episodes found that majority of PD subjects were able to have their apomorphine sublingual film dose successfully titrated to an effective and tolerable level within the first 3 titrated doses. Improvement of motor function was generally consistent in apomorphine sublingual film dose groups, suggesting that apomorphine sublingual film provided an effective treatment for OFF episodes [63]. Apomorphine sublingual film has been approved for the treatment of OFF episodes in PD individuals [64].

Different studies also provided sufficient evidence that apomorphine is beneficial to non-motor symptoms of PD patients [65-69]. Apomorphine has been reported to have moderate improvements in sleep, attention, mood, apathy, fatigue, and gastrointestinal function of PD patients [68]. Trials conducted with instrumental diagnostic tests have shown that apomorphine can improve swallowing dysfunction and relieve anorectal and urination manifestations [70-72]. Several case reports have found that apomorphine has a certain effect on OFF-related pain, especially for visceral manifestations, such as acute genital pain, pelvic, and chest pain [67, 73]. Consequently, when the OFF period is accompanied by refractory pain, apomorphine might be a good option for alleviating patients’ discomfort. In addition, apomorphine treatment appears to be related to a lower incidence of emergency impulse control disorders than other DA agonists [74, 75].

Apomorphine was well tolerated by subcutaneous administration, and only mild adverse reactions, such as yawning, drowsiness, nausea, and dizziness, were observed in clinical trials [72, 76]. Skin nodules at the site of injection are a common and troubling complication that usually develops over time owing to antioxidant sodium metabisulphite in apomorphine solution. Rotation of infusion sites, application of Teflon needles and ultrasound techniques, and the adjustment of delivery through the skin to an optimum angle have been shown to be effective for improving skin nodules and thus making the infusion sites appropriate for further injections [53, 69].

#### 4.1.2 Rotigotine

Rotigotine is a non-ergot agent with high affinity for D1, D2 and D3 receptors but low affinity for D4 and D5 receptors [77]. A single rotigotine transdermal patch can provide a stable blood concentration for 24 hours through continuous transdermal release, which may increase compliance, especially for patients with dysphagia and those taking several oral medications [78].

The beneficial effects of rotigotine on motor symptoms of PD (OFF time, UPDRS-III) and the positive effects on activities of daily living (ADL, UPDRS-II) have been demonstrated in 11 clinical trials in early PD patients using rotigotine as a single therapy [79-84] or in advanced PD sufferers with a combined use of levodopa [85-90]. A post hoc analysis of more than 2000 subjects in early or advanced PD [91] and a prospective study of more than 2000 PD individuals of varying stages and symptom severity [92] also showed that rotigotine was effective for motor symptoms and ADL. Several open-label extension clinical trials have also found improvements in motor symptoms with rotigotine lasting up to 6 years [93-96]. A few clinical trials have investigated the efficacy of rotigotine on motor complications, showing a low incidence of dyskinesia during long-term use of rotigotine, and dyskinesia is usually “non-disabling” or “mildly disabling [93, 94, 97].

Different studies have demonstrated that rotigotine is effective in improving non-motor manifestations of PD. Clinical studies have shown that transdermal rotigotine therapy has a significant advantage over placebo for the cure of nocturnal sleep disorders [98, 99]. A post hoc analysis further confirmed the beneficial findings for sleep, especially in terms of “difficulty falling asleep,” “feeling tired and sleepy in the morning,” or “being uncomfortable in bed due to immobility” [100]. A randomized, double-blind study on 42 PD patients with sleep disorders also found that rotigotine significantly improved sleep efficiency and continuity by promoting sleep stability and increasing rapid eye movement (REM) sleep quantity [101]. A randomized clinical study on 287 PD subjects found that rotigotine showed a beneficial effect on general pain in PD patients, and the finding was further confirmed by a post hoc analysis [99, 102]. A randomized pilot study involving 68 PD subjects also supported the beneficial effects of rotigotine on fluctuation-related pain in PD patients [103]. Rotigotine has also been found to be efficacious for the improvement of dysphagia, neuropsychiatric symptoms, and quality of life in PD patients [104-107].

Rotigotine was found to be efficacious and well tolerated in PD, especially for those in the advanced stage with dysphagia. Application site reaction is the most common adverse effect of rotigotine, and other adverse reactions include nausea and drowsiness [78]. Most of these adverse effects are mild to moderate in intensity and mostly subside rapidly after the withdrawal of the rotigotine transdermal patch [108].

### 4.2 D2 Family Agonists

#### 4.2.1 Pramipexole

Pramipexole is a selective DA agonist that primarily acts on D2/D3 receptors. Its binding capacity for the D3 receptor is 7-10 times higher than that for the D2 receptor [109, 110]. Both preparations of pramipexole sustained release and pramipexole immediate release are safe and effective for the improvement of motor dysfunction and ADL in early PD individuals and reduce the daily total OFF period for advanced PD sufferers [111-113]. In 2017, a clinical study on 136 PD individuals showed that a sustained-release compound containing a small dose of pramipexole and resagiline (P2B001) had significant clinical efficacy and a good safety profile, thus offering a promising treatment option for early PD patients. As adjunctive therapy to levodopa, the P2B001 treatment group showed significant improvement in ADL scores, responder analysis, UPDRS total score, and quality of life indicators compared to placebo [114].

Pramipexole immediate release has been found to be effective for depressive symptoms of PD patients [115]. Recently, a meta-analysis involving 1789 PD subjects also confirmed the therapeutic efficacy of pramipexole for depression in PD patients [116]. Pramipexole was recommended when PD patients were complicated with depression according to Movement Disorders Society guidelines [117]. A clinical trial of 119 PD individuals troubled with sleep disturbances found that both preparations of pramipexole sustained release and pramipexole immediate release were effective and well tolerated in improving nocturnal symptoms of PD patients troubled with sleep disorders [118]. In 2021, A pilot study on 98 patients also found that pramipexole sustained release and pramipexole immediate release showed similar benefits and safety for the treatment of nocturnal symptoms in Chinese advanced PD patients [119]. A small clinical trial of 10 subjects reported that a low dose of pramipexole improved antipsychotic-induced parkinsonism, and the study also observed an improvement in psychiatric symptoms among people with schizophrenia. However, the preliminary results from this study need to be further validated in a prospective study with a larger sample [120].

The safety profile did not differ between pramipexole immediate release and pramipexole sustained release, with sleep attacks, somnolence, nausea, postural hypotension, dizziness, and stomach discomfort being the most common adverse effects in early PD patients and dyskinesia in advanced PD sufferers [121]. A case report also found a loss of color vision during long-term therapy of pramipexole [122]. Studies reported that pramipexole was significantly associated with the development of heart failure, especially in the first few months of treatment and in elderly patients [123, 124]. Careful cardiology assessment is recommended before and during DA agonist treatment, especially in patients taking pramipexole.

#### 4.2.2 Ropinirole

Ropinirole primarily stimulates D2 and D3 receptors and has little affinity for D1, adrenergic and serotonergic receptors [125]. Ropinirole can be used as a monotherapy for early PD or in combination with levodopa for the management of advanced PD [126]. A randomized clinical study involving 161 early PD subjects found that ropinirole 24-hour sustained release and ropinirole immediate release showed similar efficiency in improving UPDRS motor score in early PD patients [127]. A clinical study with a total of 208 PD subjects found that ropinirole sustained release prominently delayed the occurrence of dyskinesia in early PD patients not fully controlled by levodopa [128]. Clinical investigations reported that ropinirole sustained release was more efficient in alleviating OFF time in advanced PD compared to placebo and superior to ropinirole immediate release in maintaining an improvement of daily OFF period [129, 130]. In 2020, a randomized clinical study on 587 advanced PD individuals investigated the safety and efficiency of a once-daily ropinirole patch as adjunctive therapy to levodopa for the treatment of PD patients. The results showed that once-daily application of the ropinirole patch was as effective as ropinirole sustained release tablets in the treatment of advanced PD patients and without serious safety concerns [131]. More recently, a long-term study on 158 PD subjects found that a once-daily ropinirole patch showed long-term safety and efficacy for 52 weeks as adjunctive therapy to levodopa in the treatment of PD patients. Most common adverse reaction was mild to moderated application site skin reactions that were well tolerated [132]. Taken together, ropinirole patch might be a novel treatment option for PD patients.

Limited data are available on ropinirole for non-motor symptoms of PD. A post-marketing trial involving 327 patients with early and advanced PD showed improvement in anxiety and depression symptoms after 12-14 weeks of ropinirole treatment [133]. A post hoc analysis involving 787 PD subjects found that prolonged ropinirole release may alleviate nocturnal symptoms of advanced PD patients, and the subgroups with PD Sleep Scale (PDSS) scores > 100 showed significant therapeutic benefits on overall sleep quality at night [134]. A study involving 33 PD patients found that prolonged release of ropinirole alleviated daytime sleepiness and improved subjective sleep quality compared to an immediate release of ropinirole, possibly attributed to a more stable plasma concentration [135].

The reported adverse reactions of ropinirole include hypotension, orthostatic hypotension, dizziness, and nausea. Ropinirole has also been found to be related to sinoatrial node dysfunction in PD patients. Excessive somnolence and sleep attacks were also reported in clinical tests and should be monitored carefully [127, 135].

#### 4.2.3 Piribedil

Piribedil is a selective DA agonist with a strong affinity for D2/D3 receptors. It also has a low affinity for α2-adrenergic and 5-Hydroxytryptamine (5-HT) receptors [136, 137]. Piribedil has been shown to be efficacious as a monotherapy or adjunct therapy with levodopa in early PD patients without motor fluctuations [138]. In 2010, a randomized, cross-over study involving 30 advanced PD patients found that a novel oro-dispersible sublingual formulation of piribedil (S90049) as adjunctive therapy to levodopa was much better than placebo in improving UPDRS-III score and switching advanced PD patients with motor fluctuations from OFF state to ON state [139]. However, there was no further study on S90049 for the treatment of PD.

Piribedil has also been found to be effective for non-motor symptoms of PD patients. Piribedil is the only preparation that has been established to be efficacious for apathy and should be taken into account when apathy occurs [140]. The effect of piribedil on daytime sleepiness has been demonstrated in randomized controlled trials [141, 142]. In conclusion, piribedil is a practical option for PD patients with apathy and excessive daytime sleepiness.

## 5 DA AGONISTS UNDERGOING CLINICAL TRIALS FOR PD

Although the D2/D3 agonists have been well characterized, it can be concluded from the clinical and preclinical investigations that targeted activation of the D1 receptor might be a promising therapeutic strategy for PD. In addition, targeted activation of the D1 receptor may also alleviate some of the problems related to existing therapies. The development of selective D1 agonists has been limited owing to the poor oral bioavailability, short half-life period, and significant changes in cardiovascular parameters. So far, new studies on selective D1 agonists for the treatment of PD have not been reported for more than 20 years. Recently, several new D1/D5 partial agonists have been investigated for the management of PD.

### 5.1 PF-06412562

PF-06412562 is a moderately potent DA agonist with a highly selective affinity for D1/D5 receptors. A randomized placebo-controlled study on 38 PD patients who received PF-06412562 found that the key exploration endpoint for changes in Unified PD Rating Scale part III (UPDRS-III) score was consistent across treatment groups compared to baseline and that improvements in UPDRS-III score were clinically significant. All adverse events were well tolerated, including nausea, vomiting, and fatigue, with no significant changes in cardiovascular parameters [143]. The results showed that PF-06412562 as a selective D1/D5 partial agonist is an effective and safe adjunctive therapy to levodopa for the treatment of PD. However, given the small number of subjects in this clinical trial, the safety and effectiveness of PF-06412562 need to be further explored in multicenter studies with larger sample sizes. In 2020, a clinical study involving 6 advanced PD patients found that PF-06412562 was well tolerated in a small cohort of advanced PD individuals [144]. Owing to the fact that PF-06412562 has the potential to provide benefits for advanced PD individuals, future research works are warranted to include larger sample sizes, longer periods of treatment, and patient-centered assessments of meaningful outcomes.

### 5.2 PF-06649751

PF06649751 is a novel compound with high selectivity for D1/D5 receptors and a non-catechol structure [145]. In 2018, two short-term phase I clinical trials of 63 PD individuals were conducted to evaluate the safety, tolerability, pharmacokinetics, and pharmacodynamics of PF-06649751 as first-line therapy for PD patients. The results showed that multiple doses of PF-06649751 up to 25 mg were safe and well tolerated, and single doses of PF-06649751 maximum to 9 mg also showed good safety and tolerability in the treatment of PD. Reported adverse effects were mild to moderate headaches, nausea, and vomiting, with no serious side effects [145]. The findings suggested that PF-06649751 deserves further studies as a treatment for PD. In 2020, a phase II clinical trial involving 47 PD patients explored the safety and effectiveness of PF-06649751 in early PD patients [146]. The study found that the UPDRS-III score was significantly improved in the PF-06649751 group compared to the placebo. No serious adverse reactions associated with orthostatic hypotension were found, such as dizziness, syncope, or vomiting. None of the subjects met the criteria for significant changes in the electrocardiogram. The research indicated that PF-06649751 had a certain effect on improving motor function as first-line therapy to early PD patients and was generally well tolerated [146].

### 5.3 PF-06669571

PF-06669571 is a novel formulation with a non-catechol structure that primarily targets the D1 receptor and has been found to be effective in preclinical models of PD [147]. The safety, tolerability, pharmacokinetics, and pharmacodynamics of PF-06669571 were evaluated in phase I clinical study on 19 PD patients taking a stable dose of levodopa. The results showed that PF-06669571 was safe and well tolerated without obvious safety problems. However, the pharmacodynamic endpoint failed to achieve the pre-specified standard for significant improvements, suggesting that PF-06669571 has a limited therapeutic effect on PD-related symptoms [147]. The time course of the pharmacological action of PF-06669571 needs further study to provide conclusive evidence.

In conclusion, selective dopamine D1/D5 agonists may be a new direction for the treatment of PD. However, the efficacy, tolerance, and pharmacodynamics of the potential D1/D5 agonists need to be further investigated in multicenter studies with larger sample sizes.

## 6 CLASS-RELATED SIDE EFFECTS

Despite the advantages of DA agonists, post-marketing studies on DA agonists also emphasized the development of important long-term adverse events, including dopamine agonists withdrawal syndrome (DAWS) [148, 149] and impulse control disorders (ICDs) [150], which greatly limit their clinical applications. Other factors, such as pharmacokinetic and pharmacodynamic properties, clinical features of patients, and concomitant medications, should also be considered when selecting a specific DA agonist to provide a more patient-tailored therapeutic choice.

### 6.1 Impulse Control Disorders (ICDs)

ICD is a complication that needs special attention in the diagnosis and treatment of PD. A total of 5% of PD sufferers have one or more clinical symptoms of ICD, and the main clinical manifestations include pathological gambling, binge eating, hypersexuality, and compulsive shopping [151, 152]. The occurrence of ICD is associated with many factors, such as the younger age of PD onset, genetic factors, personality characteristics, drug abuse, and family history. In addition, treatment with a high dosage of levodopa (equivalent dose > 450 mg/d), especially DA agonists, was strongly associated with ICD [153]. Dopamine D3 receptor agonists (such as pramipexole and ropinirole) have a comparatively high risk of ICDs [154].

In 2014, a multicenter clinical study conducted in Spain reported that oral treatment of DA agonists was found to be strongly related to the risk of ICDs compared to transdermal rotigotine, with an incidence rate of 42% and 19%, respectively [155]. These findings were further confirmed by a European multicenter survey of 425 subjects. The study found that the incidence of ICDs in the rotigotine patch group was much lower than in any other evaluated DA agonists group, except for pramipexole extended release [156]. The incidence of ICDs in the pramipexole sustained release group was much lower than that in the pramipexole immediate release group [156]. The different incidence rates of ICDs between the oral treatment of DA agonists and rotigotine patch may be associated with the different routes of administration and with the resulting stability of plasma concentration [155, 156]. Subcutaneous apomorphine infusion has been reported to be associated with the regression or weakening of preexisting ICDs [157, 158]. In 2018, a longitudinal analysis with a total of 411 patients explored the dose-effect relationship between the treatment of DA agonists and ICDs [159]. Results from the study demonstrated that the cumulative prevalence of ICDs was about 46% in 5 years and was associated with prior use of DA agonists [159]. ICDs were closely linked with the treatment of DA agonists and were shown to have a dose-response relationship. The duration of DA agonists and the increase in dose were associated with ICDs [159]. ICDs gradually disappeared after the withdrawal of DA agonists.

The development of ICDs in PD individuals who received the treatment of DA agonists has led clinicians to switch to piribedil, which has been rarely reported to be associated with ICDs. However, several cases of ICD have been reported in PD individuals receiving piribedil over the past few years, so caution should be taken when prescribing this drug to PD patients with a prior history of ICD [160]. Clinical experiences suggested that ICD can be successfully controlled by reducing the dosage of DA agonists or their discontinuation. However, switching from one DA agonist to another, even with a different pharmacological property, does not always lead to the disappearance of ICD.

There has been no effective therapy for ICD. Although small clinical studies have explored the efficacy of several agents for ICD, such as atypical antipsychotics, amantadine, and selective serotonin reuptake inhibitors, there is insufficient evidence of efficacy [161]. Clinicians should first inform patients and their families of the possibility of ICDs during DA agonist therapy. Family members should pay more attention to the life of the patient and seek treatment in time in case of abnormal behavior. Clinicians ought to reduce the dosage of DA agonists gradually and observe the changes in the disease closely. In the case of withdrawal syndrome, the minimum effective dose should be restored immediately. It has been suggested that cognitive behavioral therapy may effectively control the symptoms of ICDs [162].

### 6.2 Dopamine Agonist Withdrawal Syndrome (DAWS)

DAWS is a severe, stereotyped neuropsychiatric syndrome associated with DA agonist withdrawal that is dose-dependent and can lead to clinically significant depression or social/occupational dysfunction [95]. Potential risk factors for DAWS include higher peak dosage of DA agonists and cumulative DA agonists exposure. Longer duration of DA agonists treatment and higher levodopa equivalent daily doses have also been shown to be associated with DAWS [163, 164]. DAWS has been found after the tapering of pramipexole and ropinirole, and no significant difference was found between DA agonists [149].

Currently, there are no clear diagnostic criteria and effective treatment for DAWS. A multicenter observational study on 51 PD individuals suggested that patients diagnosed with moderate to severe DAWS required counseling, psychological intervention as well as the reintroduction of DA agonists previously interrupted [163]. Strategies to prevent the occurrence of DAWS are also essential. DA agonist treatment should be used cautiously, especially for patients complicated with ICDs, and rapid withdrawal of DA agonists should also be avoided. When DA agonist therapy is reduced or discontinued, patients should be closely monitored for DAWS symptoms. It is also recommended to avoid long-term, high-dose exposure to DA agonists as they may also increase the risk of DAWS.

### 6.3 Cardiological Side Effects

At present, there are few studies on the relationship between the use of DA agonists and heart failure. To date, no studies have offered a definitive explanation for the link between the usage of DA agonists and an increased risk of heart failure. A population-based trial of 25459 PD patients explored the relevance between the use of DA agonists and the risk of heart failure in patients with PD. The results showed that the application of pramipexole was significantly associated with the development of heart failure, especially in the first few months of treatment and in elderly patients [123]. A large population-based cohort study including 26814 PD individuals found an increased incidence of heart failure in patients currently on DA agonists compared to those not on DA agonists, particularly for the use of pramipexole and cabergoline [124].

Other cardiological side effects relevant to the use of DA agonists include sinoatrial node dysfunction associated with ropinilole and first-degree atrioventricular conduct block related to rotigotine [76, 165]. Careful cardiology assessment was recommended before and during the treatment of DA agonists, especially in patients taking pramipexole.

## 7 RECOMMENDATIONS

PD patients may have different clinical responses to a DA agonist, and it is recommended to withdraw the use of DA agonist in case of troublesome adverse reactions or the absence of benefits. DA agonist therapy should be initiated at low doses and increased until beneficial clinical effects are achieved. Elderly patients are at greater risk of adverse events during DA agonists therapy, and a risk-benefit analysis should be conducted prior to initiation of DA agonists treatment according to the presence of motor complications, cognitive dysfunctions, and comorbidities [166]. Patients' living habits and preferences should also guide the clinicians in drug selection.

### 7.1 Motor Symptoms

**Fig. (2):**
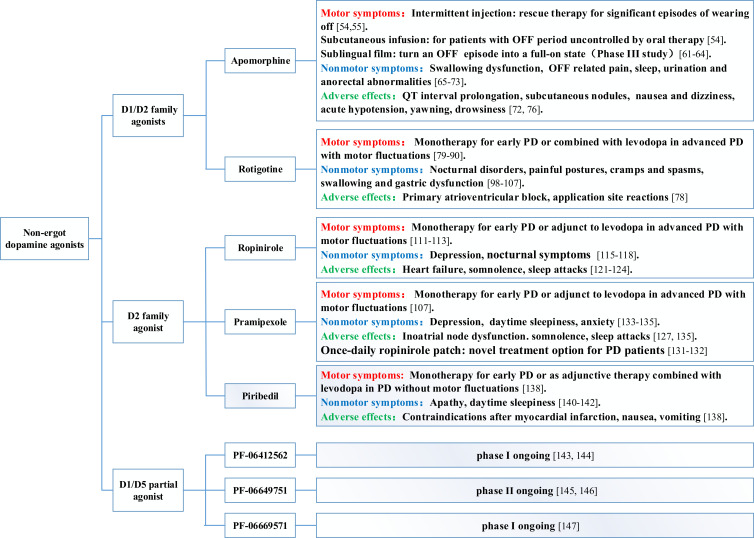
Classification, clinical indication, and adverse effects of non-ergot dopamine agonists.

Rotigotine, ropinolole, and pramipexole have presented equal clinical efficacy for the treatment of motor symptoms in PD both as a monotherapy or as adjunctive therapy combined with levodopa [167]. Intermittent injection of apomorphine is recommended for advanced PD sufferers troubled with drug-resistant OFF time and peak dose dyskinesia that cannot be fully controlled by standard oral treatment [54]. Apomorphine subcutaneous injection is recommended as rescue therapy for patients experiencing significant, unpredictable episodes of wearing off, early morning akinesia, and OFF-related pains due to its rapid speed of onset [53, 54, 168]. Apomorphine sublingual film showed clinical efficacy for OFF episodes in PD patients, which deserves further study (Fig. **2**).

### 7.2 Non-motor Symptoms

#### 7.2.1 Neuropsychiatric Symptoms

**Fig. (3):**
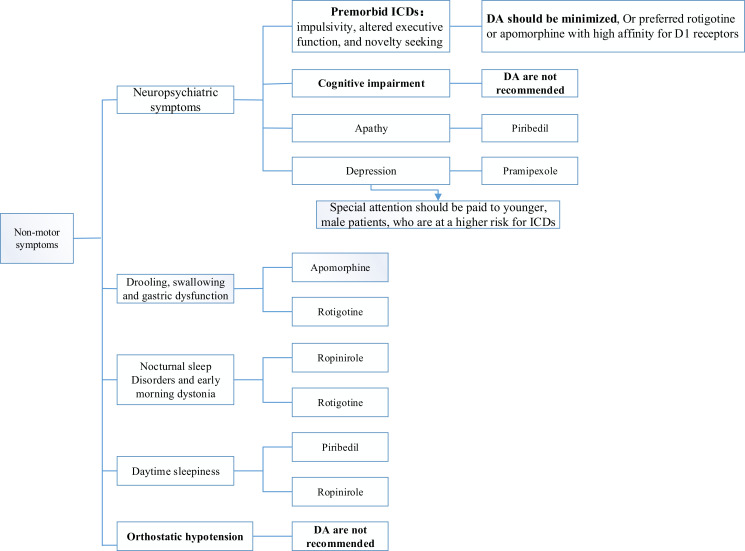
Medication selection for non-motor symptoms of PD.

Pramipexole is beneficial to control the mental complications of PD and may be a potential antidepressant [115]. Pramipexole alone or in combination with classic antidepressants is more beneficial for PD patients with depression [115]. Piribedil is the only agent that has been shown to be efficacious for the treatment of apathy in PD patients and should be considered when apathy occurs [138]. For PD patients with depressive symptoms, special attention ought to be paid to younger male PD sufferers, who are at a higher risk of ICDs. The study also found that PD patients with baseline personality traits, such as impulsivity, novelty seeking, and altered executive function, were at increased risk of ICD. Consequently, the application of DA agonists in this population should be minimized, or rotigotine or apomorphine with high affinity for D1 receptors should be preferred [169]. The occurrence of cognitive impairment should discourage the application of DA agonists as they may induce or aggravate confusion, hallucinations, and other psychiatric manifestations. The emergence of delirium, hallucinations, and confusion may require a temporary or permanent withdrawal of DA agonists along with a compensatory elevated daily dose of levodopa [117] (Figs. **2** and **3**).

#### 7.2.2 Sleep Disorders

Rotigotine and ropinirole can improve nocturnal dyskinesia and morning dystonia and promote sleep continuity, thereby improving subjective sleep quality [80, 81, 116]. Ropinirole prolonged-release can reduce daily drowsiness and lead to the disappearance of sudden onset sleep in some patients due to its more stable plasma concentration [135]. However, it has been found that sudden onset sleep, daily drowsiness, and sleep attacks are frequently related to pramipexole and, to a lesser extent, ropinirole. Given that these symptoms often appear without notice, daily sleepiness needs to be closely monitored with a specific scale [76, 170].

#### 7.2.3 Autonomic Dysfunction

Apomorphine and rotigotine should be given priority to patients with drooling, dysphagia, or obvious gastrointestinal dysfunction due to their optional administration routes and the enhancement of gastrointestinal activity mediated by the D1 receptor [171-174]. Apomorphine should be used with caution in patients with postprandial hypotension because it may cause a sharp decrease in blood pressure. Levodopa should be preferred in PD patients suffering from orthostatic hypotension (OH), as DA agonist is known to aggravate OH through vasodilation of the peripheral vascular bed [175]. Midodrine, domperidone, fludrocortisone, and droxidopa are recommended for the treatment of OH [176-178]. Excessive sweating is a common nonmotor symptom that leads to poor adhesion and reduces the therapeutic effect of rotigotine [179].

### 7.3 Concomitant Medication and Comorbidities

It is important that patients be carefully evaluated for comorbidities and concomitant medications before prescribing a specific DA agonist, as all DA agonists except pramipexole are primarily metabolized in the liver, and pramipexole is mainly excreted in urine through active renal tubular secretions [72, 77, 125, 180]. The residual DA agonist is primarily eliminated by the cytochrome P450(CYP) enzyme system in the liver. Therefore, special attention is required for PD individuals receiving the treatment of other CYP inhibitors.

Pramipexole is especially suitable for patients treated with multiple medications to minimize the onset of drug interactions. On the contrary, pramipexole should not be applied in PD individuals with advanced kidney dysfunction, and the dose must be adjusted individually in PD sufferers with mild to moderate kidney dysfunction [180]. The association between the usage of pramipexole and heart failure still needs to be fully clarified, but a risk-benefit analysis ought to be conducted before the initiation of pramipexole in older, male, high-risk patients with a history of cardiovascular disorder, hypertension, diabetes, alcoholism, smoking as well as physical inactivity [181].

## CONCLUSION

DA agonists are effective agents for the management of motor and non-motor symptoms of PD patients. The new once-daily DA agonists and transdermal preparations made the use of DA agonists easier and increased compliance of patients and adherence to treatment. Clinical studies also showed that sustained released formulations of DA agonists were superior to immediate-release formulations in alleviating OFF time. Several studies showed the efficacy of DA agonists in improving some non-motor symptoms, especially nocturnal disturbances, apathy, and depression. However, over the past few years, there have been some clinical problems with the application of DA agonists. In addition to the common side effects, such as hypotension, dizziness, nausea, and stomach discomfort related to the application of DA agonists, ICDs and DAWS are adverse reactions that clinicians should consider when using DA agonists and know how to deal with them.

In conclusion, DA agonists represent an excellent treatment option for PD patients in the early stages of the disease but also in combination with levodopa and other dopaminergic drugs for advanced PD individuals. However, not all DA agonists are exactly the same. Each of these DA agonists has a unique efficacy and safety profile because of the different pharmacokinetic and pharmacodynamic properties. It is vital for clinicians to prescribe these medications at the right timing, at the right doses, and in the right combinations with other anti-parkinsonian drugs. Individual clinical characteristics, prominent non-motor symptoms, and comorbidities should all be considered in order to optimize benefits and minimize both short-term and long-term adverse reactions. At present, the existing DA agonists have not reached the ideal state in the treatment of PD. Priority should be given to the development of a new dopaminergic compound with a rapid therapeutic effect, long duration of response, a manageable route of administration, and fewer side reactions. In the future, findings on this topic would likely help tailor the appropriate drugs for each individual patient.

## LIST OF ABBREVIATIONS

ADL: Activities of Daily Living
DA: Dopamine Agonists
DAWS: Dopamine Agonist Withdrawal Syndrome
5-HT: 5-Hydroxytryptamine
ICDs: Impulse Control Disorders
MPTP: N-methyl-4-phenyl-1,2,3,6-tetrahydropyridine
OH: Orthostatic Hypotension
6-OHDA: 6-Hydroxydopamine
PD: Parkinson's Disease
PDSS: Parkinson’s Disease Sleep Scale
UPDRS: Unified Parkinson’s Disease Rating Scale
